# Dictyopterenes A, B, C, and D from Marine Algae

**DOI:** 10.3390/molecules30193987

**Published:** 2025-10-04

**Authors:** Igor Jerković, Anja Golemac Lipovac, Dina Balaić, Stela Jokić

**Affiliations:** 1 Faculty of Chemistry and Technology, University of Split, Ruđera Boškovića 31, 21000 Split, Croatia; 2 Mediterranean Institute for Life Sciences, University of Split, Meštrovićevo šetalište 45, 21000 Split, Croatia; aglipovac@medils.unist.hr (A.G.L.); dbalaic@medils.unist.hr (D.B.); 3 Faculty of Food Technology Osijek, University of Josip Juraj Strossmayer in Osijek, Franje Kuhača 18, 31000 Osijek, Croatia; sjokic@ptfos.hr

**Keywords:** dictyopterene A, dictyopterene B, dictyopterene C, dictyopterene D, dictyotene, ectocarpene, hormosirene, marine algae

## Abstract

The review is focused on dictyopterenes A, B, C, and D found in marine algae, covering their (a) distribution; (b) methods of isolation and identification; (c) absolute configuration; and (d) biosynthesis considerations. Dictyopterenes A and B are usually present in high amounts in *Dictyopteris* spp. Dictyopterene A was found to be abundant in *D. prolifera, D. undulata*, *D. latiscula*, *D. polypodioides*, and *D. membranacea*. Dictyopterene B (hormosirene) was found as the major compound in *D. plagiogramma*, *D. australis*, *Hormosira banksii*, *D. potatorum*, *D. willana*, *D. antarctica*, *Xiphophora chondrophylla*, *X. gladiata*, *Scytosiphon lomentaria*, *Colpomenia peregrina*, and *Haplospora globosa.* Dictyopterene C (dictyotene) was a major compound in *D. undulata*, *D. prolifera*, *D. membranacea*, *Gomphonema parvulum*, *Amphora veneta*, *Phaeodactylum tricornutum*, and *D. vermicularis*. Dictyopterene D (ectocarpene) was present in *Ectocarpus siliculosus*, *Analipus japonicus*, *D. prolifera*, *D. undulata*, and *Sargassum linifolium*. The following enantiomers were found: (1*S*,2*R*)-dictyopterene A, (1*R*,2*R*)-dictyopterene B, (1*S*,2*S*)-dictyopterene B, (1*S*,2*R*)-dictyopterene B, (*R*)-dictyopterene C, and (*S*)-dictyopterene D. In marine algae, C_11_-hydrocarbons are derived from C_20_ polyunsaturated fatty acids by the oxidative cleavage via, e.g., 9-hydroperoxyicosa-(5*Z*,7*E*,11*Z*,14*Z*,17*Z*)-pentaenoic acid. An alternative biosynthetic pathway for dictyopterene A and B via the proposed intermediates (*S*)-dictyoprolenols was considered by oxidative cleavage of hydroperoxyicosatetraenoic acid.

## 1. Introduction

Marine algae volatile organic compounds (VOCs) are small, low-molecular compounds with low to moderate hydrophilicity and high vapour pressure. Among others, they have been reviewed regarding (a) their emission and roles in algae [1]; (b) the production and role of algal volatile halogenated compounds [2]; (c) the role of algae and cyanobacteria in the production and release of odorants [3]; and (d) the current understanding of algal VOC production and emission in the extreme environments [4].

The trend in marine fragrance (“sea-breeze” or “ocean-smell”) is relatively recent, and its chemistry has been mainly associated with four groups of natural organic compounds [5], including cyclic and alicyclic C_11_-hydrocarbons. According to their molecular structures, non-isoprenic C_11_-compounds can be classified into the following groups: acyclic olefins, cyclopentenes, dictyopterenes, related cyclopropanes, and cycloheptadienes. The C_11_-hydrocarbons and their derivatives have been isolated from different genera of brown algae (e.g., *Desmarestia*, *Zonaria*, *Dictyota*, *Laminaria*, *Ectocarpus*, or *Fucus* genera), and have also been detected in diatom cultures, blooms of freshwater microalgae, and higher plants [6]. However, they appear to be most abundant in brown algae of the genus *Dictyopteris* [7]. The genus *Dictyopteris* J.V. Lamouroux (from Greek *Dictyon* = network, and *Pteris* = fern) was first proposed by Lamouroux in 1809 [8] and belongs to the Dictyotales order [9] and is widely distributed in tropical, subtropical, and temperate regions. The brown algae of the genus *Dictyopteris* are odoriferous seaweeds [10] containing C_11_-hydrocarbons.

The chemical diversity, biological potential, and ecological roles of marine seaweeds of the *Dictyopteris* genus were reviewed [11,12,13,14]. Some species of this genus show a distinct phytochemistry, with specific secondary metabolites, including C_11_-hydrocarbons, sulphur compounds, quinone derivatives, terpenes, steroids, and halogenated compounds. This chemical diversity gives them interesting biological properties, including cytotoxic, antioxidant, anti-inflammatory, antimicrobial, and anti-herbivory activities [11]. In distinction from the previous reviews, the present review is, for the first time, focused entirely on dictyopterenes A, B, C, and D found in marine algae, covering their different aspects as follows: (a) distribution among marine algae; (b) methods of isolation and identification; (c) absolute configuration and the presence of enantiomers; and (d) biosynthesis considerations. The used database for search was Web of Science, and keywords were dictyopterene A, dictyopterene B, dictyopterene C, dictyopterene D, dictyotene, ectocarpene, and hormosirene.

## 2. Results

**Figure 1:**
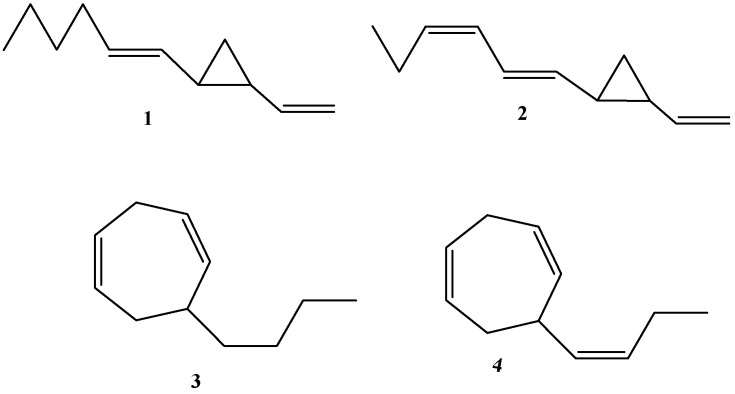
The chemical structures of dictyopterene A (**1**), dictyopterene B (**2**), dictyopterene C (**3**), and dictyopterene D (**4**).

The chemical structures of selected dictyopterenes are presented in Figure 1 as follows: dictyopterene A (*trans*-1-(*trans*-hex-1′-enyl)-2-vinylcyclopropane), dictyopterene B (*trans*-l-(*trans*,*cis*-hexa-1′,3′-dienyl)-2-vinylcyclopropane), dictyopterene C (6-butylcyclohepta-1,4-diene), and dictyopterene D (6-(*cis*-but-l’-enyl)cyclohepta-1,4-diene). Although some papers reported dictyopterenes C’ or D’ (most probably referring to their isomers), in this review only the symbols A, B, C, and D are used. In the case when the compound’s absolute configuration and correct isomer were determined, that was highlighted.

### 2.1. Distribution of Dictyopterenes A, B, C, and D in Marine Algae

Dictyopterenes A and B (also known as hormosirene) are usually present in high amounts in *Dictyopteris* spp. [15,16,17], while in the female gametes of the marine brown alga *Analipus japonicus* dictyopterene D (also known as ectocarpene) is the most abundant [18]. Dictyopterene C (also known as dictyotene) was found as the major compound in *Dictyopteris undulata* [19].

Detailed distribution of dictyopterenes A, B, C, and D in marine algae is presented in Table 1, according to the year of publication.

### 2.2. Abundance of Dictyopterene A

Dictyopterene A was isolated by preparative gas chromatography (GC) and reported as a novel hydrocarbon. The compound’s structure was determined by NMR as *trans*-1-(*trans*-1-hexenyl)-2-vinylcyclopropane by Moore, Pettus, and Doty in 1968 [20] from the essential oil of algae from the genus *Dictyopteris* and later reported among the major constituents of their essential oil (25%) [10,15,21].

The occurrence of dictyopterene A (0.24–32.9%) was also confirmed in the volatiles from *Dictyopteris prolifera* extracted by the closed-loop stripping technique without heating, and it was higher in the VOCs from the algae along the Japan sea coasts, Susa and Yoshimo, than in those from Osaka and Hikoshima [36]. The volatile compounds released from protoplasts and intact plants of *D. prolifera* from Japan were extracted by closed-looping headspace (CLHS), and dictyopterene A was found [43] with high abundance (19.08%; 18.67%).

Dictyopterene A was present in the essential oils of *D. prolifera* (63.3%), *Dictyopteris undulata* (20.9%), and *Dictyopteris* spp. (10.9%) from Japan [36]. *D. prolifera* and *D. undulata* were extracted with pentane saturated with methanol [25], and the extracts were chromatographed on an alumina column and re-chromatographed on a silica gel column. The GC-MS analysis revealed dictyopterene A in the extracts of *D. prolifera* with 65% and of *D. undulata* with 69%. Dictyopterene A was found as the major compound after simultaneous distillation extraction (SDE) in *D. prolifera* (48.9%), *D. undulata* (21.1%), *D. latiscula* (40.1%), and *Dictyopteris* sp. (10.9%) from Japan [16]. The volatile fraction of *Dictyopteris polypodioides* was prepared from the crude extract by hydrodistillation, and dictyopterene A was found (14.1%; 9.3%, respectively) [52].

Focused microwave-assisted hydrodistillation (FMAH) and hydrodistillation (HD) were applied to *Dictyopteris membranacea* from the Atlantic coast of Brittany, and GC and GC-MS revealed dictyopterene A as the major compound (32.3%—FMAH; 7.6%—HD) [47]. Supercritical carbon dioxide extraction (SC-CO_2_) and FMAH were used comparatively to isolate the volatile oils of *D. membranacea* (collected from the Mediterranean coast of Algeria) from the crude ether extract, and the major compound determined by GC-MS was dictyopterene A [54].

A minor abundance of dictyopterene A is presented in Table 1.

#### Enantiomeric Distribution of Dictyopterene A

**Figure 2:**
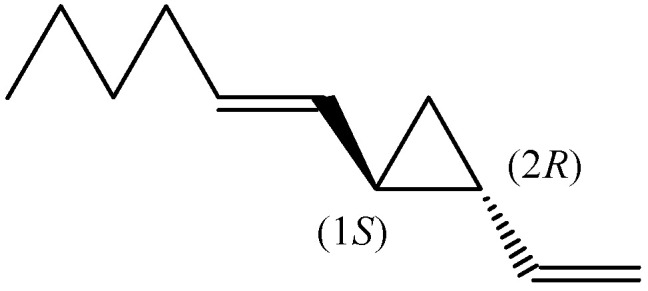
The chemical structures of (1*S*,2*R*)-dictyopterene A.

Dictyopterene A is 1-[(1*E*)-1-hexen-1-yl]-2-vinylcyclopropane, containing a vinyl and an *E*-hexenyl substituted ring with two chiral centres at positions 1 and 2 of the cyclopropane. (1*S*,2*R*)-Dictyopterene A (Figure 2) was found in the extracts from *D. prolifera* (48.9%; enantiomeric excess (e.e.) 97.7%), *D. latiscula* (40.1%; e.e. 95.1%), *D. undulata* (21.1%; e.e. 96.6%), and *Dictyopteris* spp. (10.9%, e.e. 84.2%) [16]. The extracts were obtained by digestion with pentane containing 30% methanol and fractionated on silica gel, then re-chromatographed on AgNO_3_-silica gel and subjected directly to a chiral GC analysis using the Lipodex column.

### 2.3. Abundance of Dictyopterene B

Pettus and Moore in 1970 [21] determined the chemical structure of dictyopterene B (known also as hormosirene) by NMR (*trans*-1-(*trans*,*cis*-hexa-1′,3′-dienyl)-2-vinylcyclopropane) as the major compound (50%) of the essential oil from algae of the genus *Dictyopteris* [10,21]. Dictyopterene B was isolated for the first time as the major compound (50%) from the essential oil of *Dictyopteris plagiogramma* and *Dictyopteris australis* (Hawaiian Islands) after column silver nitrate-silica gel chromatography and characterized by NMR [15].

*Hormosira banksii* is a taxonomically isolated brown seaweed endemic to Australia and New Zealand. The sperm attractant of this species has been isolated [28] and identified as hormosirene. *Hormosira* is the first organism in which a cyclopropane derivative has been found to act as a hormone in sexual reproduction. The release of the spermatozoid attractant hormosirene by the eggs of Australian fucoid *Hormosira banksii* has been determined quantitatively by a closed-loop extraction technique [41], and the total amount of hormosirene produced per egg was 2.35 pmol (0.35 ng). The occurrence of hormosirene and dictyopterene A (ratio ca. 9:1) was reported [29] in female gametes of seven additional brown algae from different systematic groups: *Durvillaea potatorum*, *Durvillaea willana*, and *Durvillaea antarctica* (Durvillaeales); *Xiphophora chondrophylla* and *Xiphophora gladiata* (Fucales); *Scytosiphon lomentaria* and *Colpomenia peregrina* (Scytosiphonales).

Dictyopterene B was present in *D. prolifera* (10%) and *D. undulata* (9%) from Japan after the extraction with pentane saturated with methanol and chromatography on an alumina column and re-chromatography on a silica gel column [25]. It was found as the major constituent of the extract of *Haplospora globosa* [30]. The steam distillation was applied to *D. prolifera*, and dictyopterene B and C were not resolved well on the used DB-1 column; their sum percentage ranged from 12.3% to 59.9% [36]. CLHS isolated the volatiles from *D. prolifera* from Japan with dictyopterene B and dictyopterene C in the protoplast (15.82%) and intact plant (16.00%) [43]. SDE was performed on *D. prolifera* from Japan provided dictyopterene B (15.6%) in the isolate [16].

Table 1 contains data about dictyopterene B minor abundance.

#### Enantiomeric Distribution of Dictyopterene B

**Figure 3:**
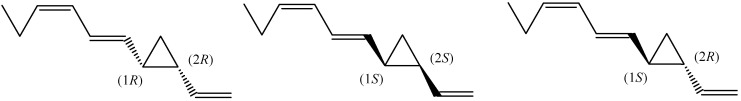
The chemical structures of (1*R*,2*R*)-, (1*S*,2*S*)-, and (1*S*,2*R*)-dictyopterene B.

Dictyopterene B contains two chiral centres at C1 and C2 of the cyclopropane ring. The inclusion chromatography on cellulose tribenzoate coated on silica resulted in the separation of (±)-hormosirene [33]. With this method, the enantiomeric excess and the absolute configuration of *Hormosira banksii*, *Durvillaea potatorum*, *Xiphophora chondrophylla*, *Dictyopteris membranacea*, *Haplospora globosa*, and *Xiphophora gladiata* were determined, and none of these species produce enantiomeric pure hormosirene [33]. The highest e.e. of (1*R*,2*R*)-dictyopterene B (Figure 3) was found in *H. banksii* (83% e.e.), followed by *D. potatorum* (52% e.e.). This isomer was present in *D. membranacea* (71% e.e.), while *H. globosa* produced opposite enantiomer (1*S*,2*S*)-dictyopterene B (83% e.e.) [33].

(1*S*,2*S*)-dictyopterene B enantiomer was discovered by the chiral HPLC analysis as a minor component in the female gametes and essential oils of Scytosiphonaceae (*S. lomentaria*, *C. bullosa*), Chordariaceae (*A. japonicus*), and Dictyotaceae (*D. prolifera* and *D. undulata*) families from Japan [39], with the highest enantiomeric composition (17.0%) in *A. japonicus*. The enantiomeric composition of (1*R*,2*R*)-dictyopterene B in female gametes and in essential oil was the following: *S. lomentaria* (97.0%), *C. bullosa* (96.5%), *A. japonicus* (83.0%), *D. prolifera* (95.0%), and *D. undulata* (96.5%). The optical purities of dictyopterene B in *S. lomentaria*, *C. bullosa*, *D. prolifera*, and *D. undulata* were much higher than that in *A. japonicus*. The amount of (1*S*,2*S*)-hormosirene (66% e.e.) was determined by a HPLC (Zorbax ODS) after the extraction (CH_2_Cl_2_-MeOH) from female gamete suspensions of *A. japonicus* [42].

(1*S*,2*R*)-dictyopterene B was found among volatile components in the essential oils from *D*. *prolifera* (15.6%, e.e. 98.9%), *Dictyopteris latiscula* (3.0%, e.e. 98.7%), *D. undulata* (2.3%, e.e. 90.1%), and *Dictyopteris* spp. (0.6%, e.e. 78.8%) [16].

### 2.4. Abundance of Dictyopterene C

Dictyopterene C is also known as dictyotene. *Dictyopteris plagiogramma* and *Dictyopteris australis* from the Hawaiian Islands contained dictyopterene C (10%) [15].

The steam distillation of *D. prolifera* provided dictyopterene B and C (their sum percentages were 12.3% to 59.9%) in the volatile fraction, but they were not resolved well on the DB-1 column [36]. The volatiles from *D. prolifera* from Japan were isolated by CLHS with a high abundance of dictyopterene B, with dictyopterene C in the protoplast (15.82%) and intact plant (16.00%) [43]. Dictyopterene C was found in the essential oils of *D. prolifera* (18.2%), *Dictyopteris undulata* (38.5%), and *Dictyopteris* sp. (10.5%) from Japan [19]. VOCs secreted by eggs from *Dictyota diemensis* were collected using the Grob–Hersch closed-loop extraction method, and dictyotene was found (31%) [37]. In *Dictyopteris membranacea*, dictyopterene C (74.1%) was identified by the closed-loop stripping technique [34].

The freshwater diatoms were cultivated in batch cultures [45]. The VOCs liberated by diatoms after initiation of the lipoxygenase reactions by NaCl treatment included unsaturated acyclic and alicyclic hydrocarbons. The VOCs stripped in a closed-loop stripping device were adsorbed on Tenax TA and analyzed using GC-MS by thermodesorption. Dictyopterene C was found in *Gomphonema parvulum*, *Amphora veneta*, and *Phaeodactylum tricornutum* [45].

*Dasycladus vermicularis* collected in Croatia in the headspace (determined by HS-SPME/GC-MS) contained dictyopterene C (8.65%; 9.34%) [57].

Dictyopterene C, as a minor constituent of marine algae, is presented in Table 1.

#### Enantiomeric Distribution of Dictyopterene C

**Figure 4:**
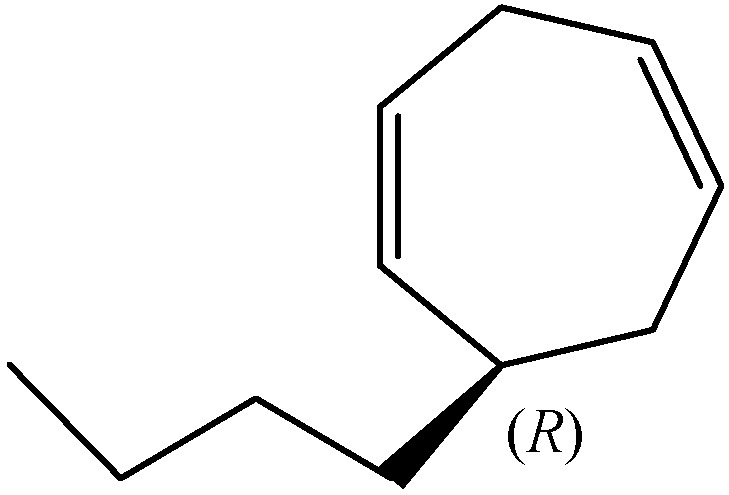
The chemical structure of (*R*)-dictyopterene C.

Dictyopterene C (6-butylcyclohepta-1,4-diene) contains one chiral centre at C6. The fraction 2 from the chromatography of the essential oil of *Dictyopteris* spp. on silver nitrate-silica gel revealed dictyopterene C as (*R*)-butylcyclohepta-1,4-diene. The ozonolysis to (*R*)-butylsuccinic acid established the configuration at C-6 as *R* [10].

(*R*)-Dictyopterene C (Figure 4) was found in the essential oil of *D. prolifera* (5.8%; e.e. 97.1%), in the oil of *D. latiscula* (2.2%; e.e. 92.3%), the oil of *D. undulata* (2.0%; e.e. 94.2%), and the oil of *Dictyopteris* spp. (0.9%; e.e. 82.7%) [16].

(+)- and (−)-Dictyotene were equally effective in stimulating sperm attraction of *D. diemensis*, and the comparison of the chemotactic response to (+)- and (−)-dictyotene revealed an apparently greater effect for the (+) enantiomer in seawater [37].

### 2.5. Abundance of Dictyopterene D

It took ca. 100 years since the initial observation to prove the existence of a female-released attractant for male gametes [60]. Another 20 years were required to determine the chemical structure of the first brown algal pheromone [22] when gynogametes (female eggs) of the brown alga *Ectocarpus siliculosus* from more than 1000 culture dishes were extracted by a stream of air passing a cold trap for condensation. After bioassay-guided chromatographic fractionation, the infrared spectrum indicated the presence of *cis*-configuration of the unsaturated side chain, and the collected compound was identified by NMR and mass spectroscopy as 6-(1(*Z*)-butenyl)-cyclohepta-1,4-diene and was named *Ectocarpus* sirenine [22], but later was revised to ectocarpene (dictyopterene D). Later, it was found by the closed-loop stripping technique and GC-MS from *E. siliculosus* [26].

The most prominent fraction of low-molecular volatile secretion products of *E. siliculosus* gametes collected on an adsorbent bed of activated carbon in a Grob–Hersch closed-loop system was identified as ectocarpene as the main compound, the pheromone bouquet of *E. siliculosus* [35]. In *Analipus japonicus* dictyopterene D was found as the major constituent by the closed-loop stripping system [42].

Furthermore, it was determined that ectocarpene was a moderately active pheromone in comparison to pre-ectocarpene (*cis*-disubstituted bis-alkenylcyclopropane: *cis*-1-alyl-2-hex-1,3-dienylcyclopropane) with ca. 10,000-fold higher activity [44]. Pre-ectocarpene was extracted at 0 °C from a dense suspension of freshly released female gametes of *E. siliculosus*, as analyzed by HPLC [44]. However, the half-life for pre-ectorapene rearrangement is significantly longer than the time required for sexual encounter in algae [44], but rapid, temperature-dependent degradation (Cope rearrangement) of the cyclopropyl compound to ectocarpene was noticed. These results suggest that systems in which cycloheptadienes were identified as pheromones or release factors should be re-examined. In addition, when identification of the volatile organic compounds relies entirely on GC methods, the rearranged products (artefacts) of the genuine cyclopropyl precursors could be detected.

Later, different studies reported the occurrence of dictyopterene D in marine algae among the major constituents of the volatile fraction. Dictyopterene D was found in the essential oil of *D. prolifera* (10.4–30.4%) from Japan [36]. CLHS from *D. prolifera* from Japan determined dictyopterene D (4.62%; 8.91%) [43]. Dictyopterene D was found in the essential oils of *D. prolifera* (15.9%), *D. undulata* (40.7%), and *Dictyopteris* sp. (20.5%) from Japan [19]. Dictyopterene D was a major constituent (20.26%) in the essential oil of *Sargassum linifolium* [49]. *Dictyota dichotoma* contained dictyopterene D (0.07–8.22%) in the headspace (HS-SPME/GC-MS) [58].

Table 1 presents the species with minor dictyopterene D abundance.

#### Enantiomeric Distribution of Dictyopterene D

**Figure 5:**
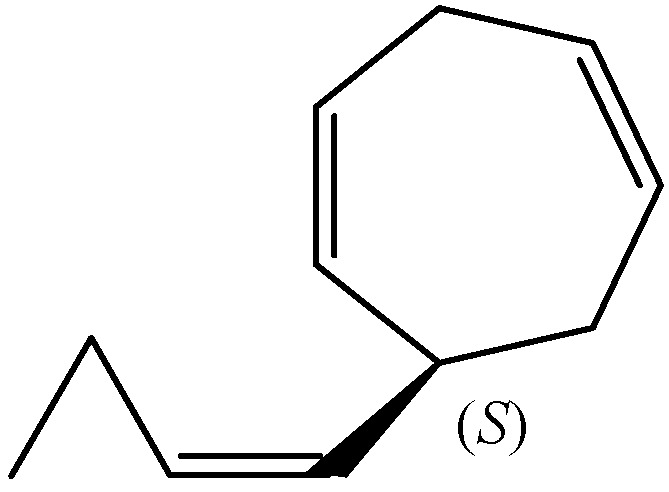
The chemical structure of (*S*)-dictyopterene D.

Dictyopterene D (6-(1(*Z*)-butenyl)-cyclohepta-1,4-diene) contains one chiral centre at C6. The absolute configuration of dictyopterene D was determined as (+)-(*S*)-6-(1(*Z*)-butenyl)-cyclohepta-1,4-diene (Figure 5) after silver nitrate-silica gel column chromatographic separation and NMR analysis of the essential oils from *Dictyopteris plagiogramma* and *D. australis* collected at the Hawaiian Islands [15]. Dictyopterene D was identical in all respects, including optical properties, with the male-attracting substance, ectocarpene, excreted by the female gametes of *E. siliculosus* [15].

Enantiomeric compositions of dictyopterene D, which cannot be separated by commercially available chiral GC or LC columns, was successfully determined by chiral GC analysis using Lipodex after side-chain reduction with hydrazine as a form of hydrogenated dictyopterene D as follows: (*S*)-dictyopterene D present with 2.2% (e.e. 99.9%) in the essential oil of *D. prolifera*, 0.1% (e.e. 99.1%) in the oil of *D. latiscula*, and 0.2% (e.e. 99.1%) in the oil of *D. undulata* [16].

### 2.6. Biosynthesis of Dictyopterenes A, B, C, and D

The structural similarities among C_11_-hydrocarbons suggest a common biosynthetic origin. In terrestrial plants, these compounds are generated from unsaturated C_12_ precursors [40] by three consecutive β-oxidations and a final oxidative decarboxylation of the resulting dodeca-3,6,9-trienoic acid [6].

In marine algae, C_11_-hydrocarbons are derived from the aliphatic terminus of C_20_ polyunsaturated fatty acids by the oxidative cleavage [40,61,62]. Female gametes of *Ectocarpus silliculosus* and *Sphacelaria rigidula*, as well as thalli of *Giffordia mitchelliae*, metabolized externally added [^2^H_n_]icosanoic acids into the hydrocarbon pheromones ectocarpene, dictyotene, and finavarrene [40]. The series of C_11_H_16_ hydrocarbons originated from *all-cis*-5,8,11,14,17-eicosapentaenoic acid, and the C_11_H_18_ compound dictyotene was produced from *all-cis*-5,8,11,14-eicosatetraenoic acid (arachidonic acid). Externally supplied [^2^H_8_]arachidonic acid can be converted into labelled 6-butylcyclohepta-1,4-diene (dictyotene) in high yield by female gametes of *E. siliculosus* [40]. Ectocarpene should arise from the aliphatic terminus of the more unsaturated eicosapentaenoic acid that was established through the deuteration pattern with the [^2^H_8_]-C_19_ carboxylic acid, which was designed as a short-chain analogue of natural eicosapentaenoic acid [40].

**Figure 6:**
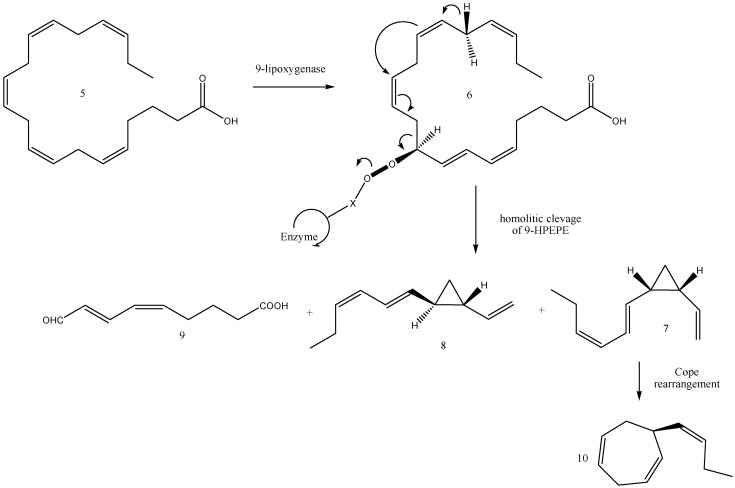
Biosynthetic concept starting from eicosapentaenoic acid (**5**): 9-hydroperoxyicosa-(5*Z*,7*E*,11*Z*,14*Z*,17*Z*)-pentaenoic acid (9-HPEPE, **6**); pre-ectocarpene (**7**), hormosirene (**8**), oxo-nonadienoic acid (**9**), (*S*)-ectocarpene (**10**).

According to the current biogenetic concept (Figure 6), 9-lipoxygenase is believed to activate, for example, the eicosanoid (e.g., eicosapentaenoic acid, **5**) as 9-hydroperoxyicosa-(5*Z*,7*E*,11*Z*,14*Z*,17*Z*)-pentaenoic acid (9-HPEPE, **6**). Subsequent cleavage of 9-HPEPE by a hydroperoxide lyase may generate C_11_ hydrocarbons from the aliphatic segment and 9-oxo-nonadienoic acid (**9**) from the fatty acid part. The same mechanistic implications, in conjunction with a different conformation of 9-HPEP at the active centre of the hydroperoxide lyase, could result in *cis*-disubstituted cyclopropane (**7**), identified as pre-ectocarpene in the *E. siliculosus* pheromone bouquet [44], with some amount of hormosirene (**8**). Furthermore, *cis*-cyclopropane (**7**) is thermolabile, and thus a subsequent spontaneous [3.3]-sigmatropic rearrangement (Cope rearrangement) is assumed to proceed [38,60] at room temperature via a *cis*–*endo* transition state to give (*S*)-ectocarpene (**10**). This hypothesis was verified by the synthesis and rearrangement reactions of thermally labile *cis*-divinylcyclopropane (**7**) and its analogues [38]. For instance, the Cope rearrangement of **7** occurred spontaneously at ambient temperatures to afford **10**. Surprisingly, comparative biological assays of *E. siliculosus* revealed that the unstable *cis*-cyclopropane **7** was much more active than the stable cycloheptadiene (**10**). The above enzymes can principally act on all types of naturally occurring eicosanoids and, thus, produce the whole variety of cyclohepta-1,4-dienes and cyclopropanes which are currently known as gamete-releasing and/or gamete-attracting pheromones of brown algae.

**Figure 7:**
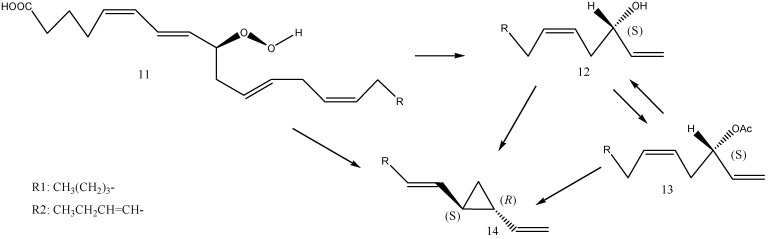
Part of the hypothetical biosynthetic pathway of dictyopterene A (14, R = R1), dictyopterene B (14, R = R2): 11-(9*S*)-hydroperoxyicosatetraenoic acid (HPITE, R = R1), 11-(9*S*)-hydroperoxyicosapentaenoic acid (HPIPE, R = R2), 12-dictyoprenol (R = R1), 12-neodictyoprolenol (R = R2), 13-dictyoprolene A (R = R1), 13-dictyoprolene B (R = R2).

An alternative biosynthetic pathway for (1*S*,2*R*)-dictyopterene A and (1*S*,2*R*)-dictyopterene B via the proposed intermediates (*S*)-dictyoprolenols (dictyoprolenol and neodictyoprolenol) was also investigated [16]. *D. prolifera* was incubated with synthetic (±)-dictyoprolenol and (±)-neodictyoprolenol as substrates, and the product content and enantiomeric composition were analyzed by GC. Added (*S*)-enantiomers in racemic substrates were selectively consumed, and a significant increase in dictyopterenes (1*S*,2*R*)-dictyopterene A and (*S*)-dictyopterene D was observed. Based on the obtained result, (*S*)-dictyoprolenol and (*S*)-neodictyoprolenol are assumed to be the possible biosynthetic intermediates for dictyopterenes. Hombeck et al. [46] reported a possible biosynthetic pathway of dictyopterene A in *Gomphonema parvulum* by oxidative cleavage of (9*S*)-hydroperoxyicosatetraenoic acid [(9*S*)-HPITE]. From (9*S*)-hydroperoxides such as (9*S*)-HPITE and (9*S*)-hydroperoxyicosapentaenoic acid [(9*S*)-HPIPE], dictyopterenes via dictyoprolenols and dictyoprolenes might be formed by stereo-specific shifting of the hydroxy group at C-9 to C-12 via a six-membered ring, as shown in Figure 7.

In addition, photochemical divinylcyclopropane trans-cis-isomerization provided strong evidence for possible in vivo photochemical steps of cis-trans-isomerization of diphenylcyclopropane during the trans-divinylcyclopropane-cycloheptadiene rearrangement [63].

## 3. Conclusions

The present review summarizes dictyopterenes A, B, C, and D found in marine algae for the first time, covering different aspects: (a) distribution among marine algae; (b) methods of isolation and identification; (c) absolute configuration and the presence of enantiomers; and (d) biosynthesis considerations. These dictyopterenes are important molecules as signalling substances (e.g., attractants, pheromones) and contribute to the ocean and beach smell.

Dictyopterenes A and B (hormosirene) are usually present in high amounts in *Dictyopteris* spp., but not exclusively. Dictyopterene A was found to be abundant in *D. prolifera*, *D. undulata*, *D. latiscula*, *D. polypodioides*, and *D. membranacea*. Dictyopterene B (hormosirene) was found as the major compound in *D. plagiogramma*, *D. australis*, *H. banksii*, *D. potatorum*, *D. willana*, *D. antarctica*, *X. chondrophylla*, *X. gladiata*, *S. lomentaria*, *C. peregrina*, and *H. globosa.* Dictyopterene C (dictyotene) was a major compound in *D. undulata*, *D. prolifera*, *D. membranacea*, *G. parvulum*, *A. veneta*, *P. tricornutum*, and *D. vermicularis*. Dictyopterene D was abundantly present in *E. siliculosus*, *A. japonicus*, *D. prolifera*, *D. undulata*, and *S. linifolium*. Since dictyopterenes A, B, C, and D were not found only in *Dictyopteris* spp., they cannot be considered specific chemical biomarkers of this genus.

The presence of exact enantiomers of dictyopterenes A, B, C, and D has been investigated rarely, and this gap can be researched further, particularly for dictyopterenes A and B that contain 2 chiral centres. (1*S*,2*R*)-Dictyopterene A was found in *D. prolifera*, *D. latiscula*, *D. undulata*, *and Dictyopteris spp.* The highest e.e. of (1*R*,2*R*)-dictyopterene B was found in *H. banksii*, followed by *D. potatorum*. This isomer was present in *D. membranacea*, while *H. globosa* produced opposite enantiomer (1*S*,2*S*)-dictyopterene B. (*R*)-dictyopterene C with high e.e. was identified in *D. prolifera*, *D. latiscula*, and *D. undulata*. (*S*)-dictyopterene D was present with e.e. 99.9% in *D. prolifera*, *D. latiscula*, and *D. undulata*.

The methods used for the isolation of dictyopterenes A, B, C, and D include distillation (e.g., hydrodistillation (HD), simultaneous distillation extraction (SDE), focused microwave-assisted hydrodistillation (FMAH)), extraction (e.g., solvent extraction, supercritical CO_2_ (SC-CO_2_) extraction), and headspace methods (e.g., closed-loop stripping technique, headspace solid phase microextraction), while their identification is made in the beginning by NMR and later by GC-MS or GC. For the determination of enantiomers, the chiral columns were used for GC or HPLC. There is potential to use the chiral analysis more abundantly for the determination of the presence of exact enantiomers in different marine algae.

In marine algae, C_11_-hydrocarbons are derived from the aliphatic terminus of C_20_ polyunsaturated fatty acids by oxidative cleavage, but the biosynthesis studies of dictyopterenes A, B, C, and D by labelled fatty acids are not so abundant. In contrast, C_11_ hydrocarbons from terrestrial plants are generated from unsaturated C_12_ precursors. According to the current biogenetic concept, 9-lipoxygenase is believed to activate, for example, the eicosanoid (9-HPEPE). Subsequent cleavage of 9-HPEPE by a hydroperoxide lyase may generate C_11_ hydrocarbons. Formed *cis*-cyclopropane is thermolabile, and thus a subsequent spontaneous Cope rearrangement is assumed to proceed at room temperature to produce (*S*)-ectocarpene. The above enzymes can principally act on all types of naturally occurring eicosanoids and, thus, produce the whole variety of cyclohepta-1,4-dienes and cyclopropanes. An alternative biosynthetic pathway for dictyopterene A and B via the proposed intermediates (*S*)-dictyoprolenols was considered by oxidative cleavage of hydroperoxyicosatetraenoic acid.

## Figures and Tables

**Table 1:** Distribution and abundance (GC peak area percentages unless indicated otherwise) of dictyopterenes A, B, C, and D in marine algae.

Species		Method of Isolation and Identification	Dictyopterenes Abundance	Reference
1.	*Dictyopteris plagiogramma* (Montagne) Vickers	short-path distillation; preparative gas chromatography, nuclear magnetic resonance (NMR)	dictyopterene A	Moore et al., 1968 [20]
2.	*Dictyopteris australis* (Sonder) Askenasy			
3.	*Dictyopteris* spp.	essential oil; gradient chromatography on 25% silver nitrate-silica gel followed by NMR	dictyopterene A (25%) dictyopterene B (50%)	Pettus & Moore, 1970 [21]
4.	*Dictyopteris* spp.	essential oil; chromatography on 25% silver nitrate-silica gel followed by GC and NMR	dictyopterene A and dictyopterene B are the major constituents; dictyopterene C	Moore & Pettus, 1971 [10]
5.	*Ectocarpus siliculosus* (Dillwyn) Lyngbye	the use of a stream of purified air and condensation; gas chromatography and mass spectrometry (GC-MS), NMR	dictyopterene D	Müller et al., 1971 [22]
6.	*Dictyopteris plagiogramma* (Montagne) Vickers *Dictyopteris australis* Sonder	chromatography of the essential oil on silica impregnated with silver nitrate; GC	dictyopterene A (around 25%) dictyopterene B (around 50%) dictyopterene C dictyopterene D	Moore et al., 1974 [15]
7.	*Dictyopteris prolifera* (Okamura) Okamura	hexane-soluble fraction of the acetone extract was chromatographed on silica gel and preparative thin layer chromatography (TLC) on silica gel; NMR	dictyopterene A (yield 0.003%) dictyopterene B (yield 0.001%)	Yamada et al., 1979 [23]
8.	*Laminaria digitata* (Hudson) J.V.Lamouroux	closed-loop stripping technique; GC-MS	dictyopterene D	Müller et al., 1979 [24]
9.	*Dictyopteris prolifera* (Okamura) Okamura	extraction with pentane saturated with methanol; GC-MS	dictyopterene A (65%) dictyopterene C (3%) dictyopterene B (10%)	Kajiwara et al., 1980 [25]
10.	*Dictyopteris undulata* Holmes		dictyopterene A (69%) dictyopterene C (5%) dictyopterene B (9%)	
11.	*Ectocarpus siliculosus*	closed-loop stripping technique; GC-MS	dictyopterene D	Müller et al., 1980 [26]
12.	*Ectocarpus siliculosus* (Dillwyn) Lyngbye	closed-loop stripping technique; GC-MS	dictyopterene D (major compound)	Müller & Gassmann, 1980 [26]
13.	*Dictyota dichotoma* (Hudson) Lamour	extraction procedure by closed-loop technique; GC-MS	dictyopterene C	Müller et al., 1981 [27]
14.	*Hormosira banksii* (Turner) Decaisne	closed-loop stripping technique; GC-MS	dictyopterene B	Müller et al., 1984 [28]
15.	*Durvillaea potatorum* (Labillard) Aresch. *Durvillaea willana* Lindauer *Durvillaea antarctica* (Chamisso) Hariot *Xiphophora chondrophylla* (R. Brown ex Turn.) Mont. ex Harv. *Xiphophora gladiata* (Labillard) Mont. ex Kjellm. *Scytosiphon lomentaria* (Lyngb.) C. Ag. *Colpomenia peregrina* (Sauv.) Hareel	closed-loop stripping technique; GC-MS	dictyopterene B and dictyopterene A (ratio ca. 9:1)	Müller et al., 1985 [29]
16.	*Haplospora globosa* Kjellman	extraction procedures for low-molecular hydrophobic substances; GC	dictyopterene B (major compound) dictyopterene D	Kuhlenkamp & Müller, 1985 [30]
17.	*Dictyopteris prolifera* (Okamura) Okamura	the hexane-soluble fraction of the acetone extract was chromatographed on silica gel; NMR	dictyopterene A (yield 0.0021%) dictyopterene B (yield 0.0019%)	Yamada et al., 1986 [31]
18.	*Skeletonema costatum* (Greville) Cleve	purging with ultrapure air in a cold trap; GC	dictyopterene D (mainly in *S. costatum*)	Derenbach & Pesando (1986) [32]
19.	*Lithodesmium undulatum* Ehrenberg			
20.	*Hormosira banksii* (Turner) Decaisne *Durvillaea potatorum* (Chamisso) Hariot *Xiphophora chondrophylla* (R. Brown ex Turn.) Mont. ex Harv. *Xiphophora gladiata* (Labillard) Mont. ex Kjellm. *Dictyopteris membranacea* (Stackh.) Batt. *Haplospora globosa* Kjellman	inclusion chromatography	dictyopterene B	Boland et al., 1987 [33]
21.	*Dictyopteris membranacea* (Stackh.) Batt.	closed-loop stripping technique; GC-MS	dictyopterene A (2.2%) dictyopterene B (2.3%) dictyopterene C (74.1%) dictyopterene D (13.4%)	Boland et al., 1987 [34]
22.	*Ectocarpus siliculosus* (Dillwyn) Lyngbye	Grob–Hersch closed-loop extraction; GC	dictyopterene D (17.4%)	Muller and Schmidt, 1988 [35]
23.	*Dictyopteris prolifera* (Okamura) Okamura	close-looping headspace (CLHS) procedure on charcoal; GC-MS	dictyopterene A (0.24–32.9%) dictyopterene D (10.4–30.4%) dictyopterene B + C (3.14–59.9%)	Kajiwara et al., 1989 [36]
24.	*Dictyota diemensis* Kützing	Grob–Hersch closed-loop extraction method; GC-MS	dictyopterene C (31%) dictyopterene D (0.5%) dictyopterene A (0.5%)	Philips et al., 1990 [37]
25.	*Cutleria multifida* (Smith) Greville	VOCs were entrapped on charcoal filters using air circulation in a closed system; GC-MS	dictyopterene C dictyopterene D	Keitel et al., 1990 [38]
26.	*Scytosiphon lomentaria* (Lyngbye) Link	steam distillate; GC, GC-MS	dictyopterene A (0.26%) dictyoptercne D (0.13%) dictyopterene C (0.32%)	Kajiwara et al., 1991 [39]
27.	*Scytosiphon lomentaria* (Lyngbye) Link	activated carbon fibre in a closed-loop stripping system for VOCs; female gametes suspension extracted with CH_2_Cl_2_; HPLC on chiral column	dictyopterene B	
28.	*Colpomenia bullosa* (D.A.Saunders) Yamada			
29.	*Analipus japonicus* (Harvey) M.J.Wynne			
30.	*Dictyopteris prolifera* (Okamura) Okamura			
31.	*Dictyopteris undulata* Holmes			
32.	*Ectocarpus siliculosus* (Dillwyn) Lyngbye	closed-loop extraction; GC-MS	dictyopterene C Dictyopterene D	Stratmann et al., 1992 [40]
33.	*Hormosira banksii* (Turner) Decaisne	closed-loop extraction technique with activated carbon filter and eluted with dichloromethane; GC	dictyopterene B	Maier & Clayton, 1993 [41]
34.	*Analipus japonicus* (Harvey) M.J.Wynne	closed-loop stripping system; GC, GC-MS, chiral HPLC	dictyopterene B: dictyopterene D: dictyopterene C (ratio 11:87:2)	Kodama et al., 1993 [42]
35.	*Dictyopteris prolifera* (Okamura) Okamura	closed-looping headspace (CLHS) procedure for isolation from protoplasts and intact plant; GC, GC-MS	dictyopterene A (19.08%; 18.67%) dictyopterene B + C (15.82%; 16.00%) dictyopterene D (4.62%; 8.91%)	Fujimura et al., 1994 [43]
36.	*Ectocarpus siliculosus* (Dillwyn) Lyngbye	extraction with MeOH and CH_2_Cl_2_; HPLC	dictyopterene D	Boland et al., 1995 [44]
37.	*Gomphonema parvulum* (Kützing) Kützing	VOC stripped in a closed-loop stripping device and adsorbed on Tenax TA and subsequently transferred to GC-MS by thermodesorption; GC-Fourier transform infrared spectroscopy (FTIR)	dictyopterene A dictyopterene C	Wendel & Jüttner, 1996 [45]
38.	*Amphora veneta* Kützing		dictyopterene C	
39.	*Phaeodactylum tricornutum* Bohlin		dictyopterene C	
40.	*Dictyopteris prolifera* (Okamura) Okamura	essential oil; GC-MS; chiral GC	dictyopterene A (63.3%), C (18.2%), D (15.9%)	Kajiwara, 1997 [19]
41.	*Dictyopteris undulata* Holmes		dictyopterene A (20.9%), C (38.5%), D (40.7%)	
42.	*Dictyopteris divaricata* (Okamura) Okamura		dictyopterene C (trace) dictyopterene D (trace)	
43.	*Dictyopteris* spp.		dictyopterene A (10.9%), C (10.5%), D (20.5%)	
44.	*Gomphonema parvulum* (Kützing) Kützing	solid phase microextraction (SPME); chiral GC-MS	dictyopterene A	Hombeck et al., 1999 [46]
45.	*Dictyopteris prolifera* (Okamura) Okamura	SDE (simultaneous distillation extraction), solvent extraction with pentane solution with 30% of ethanol; chromatography on silica gel and then on AgNO_3_-silica gel; GC, GC-MS, chiral GC	dictyopterene A (48.9%), B (15.6%), C (5.8%), D (2.2%)	Yamamoto et al., 2001 [16]
46.	*Dictyopteris undulata* Holmes		dictyopterene A (21.1%), B (2.3%), C (2.0%), D (0.2%)	
47.	*Dictyopteris latiscula* (Okamura) Okamura		Dictyopterene A (40.1%), B (3.0%), C (2.2%), D (0.1%)	
48.	*Dictyopteris* sp.		Dictyopterene A (10.9%), B (0.6%), C (0.9%), D (trace)	
49.	*Dictyopteris membranacea* (Stackhouse) Batters	focused microwave-assisted hydrodistillation (FMAH) and hydrodistillation (HD); GC and GC-MS	dictyopterene A (32.3%—FMAH; 7.6%—HD) dictyopterene C (3.3%—FMAH; 0.8%—HD)	El Hattab et al., 2002 [47]
50.	*Padina pavonia* (L.) Gaill *Hydroclathrus clathratus* (C. Agardh)	SDE; GC-MS	dictyopterene D (3.22%) dictyopterene A (0.60%)	Awad et al., 2009 [48]
51.	*Sargassum asperifolium* Hering & G. Martens ex J. Agardh	SDE; GC-MS	dictyopterene D (2.02%), dictyopterene C (0.32%)	Matloub et al., 2012 [49]
52.	*Sargassum dentifolium* (Turner) C. Agardh		dictyopterene D (4.63%), dictyopterene A (1.02%)	
53.	*Sargassum linifolium* C. Agardh		dictyopterene D (20.26%) dictyopterene A (3.18%)	
54.	*Halopteris filicina* (Grateloup) Kutzing	headspace solid-phase microextraction (HS-SPME), GC, GC-MS	dictyopterene D (1.9%) and C (0.7%)	Jerković et al., 2018 [50]
55.	*Flabellia petiolata* (Turra) Nizamuddin		dictyopterene D (7.4%) and C (0.7%)	
56.	*Padina pavonica* (Linnaeus) Thivy	HS-SPME, HD; GC, GC-MS	dictyopterene A (0.87–1.27%) dictyopterene D (1.15%)	Jerković et al., 2019 [51]
57.	*Dictyopteris polypodioides* (A.P. De Candolle) J.V. Lamouroux	essential oil (EO), steam distillation of diethyl ether extract (volatile fraction, VF), HD; GC-FID, GC-MS	dictyopterene A (14.1% in VF, 9.3% in EO) dictyopterene B’ (1.1% in VF, 0.4% in EO) dictyopterene C (trace in VF, 1.6% in EO) dictyopterene D (1.8% in VF, 0.2% in EO)	Riad, 2020 [52]
58.	*Polysiphonia senticulosa* Harvey	HS-SPME; GC–MS	dictyopterene D (0.95–1.74%)	Wang et al., 2021 [53]
59.	*Dictyopteris membranacea* (Stackhouse) Batters	FMAHD, supercritical CO_2_ (SC-CO_2_) extraction of crude diethyl ether extract; GC-MS	dictyopterene A (major compound)	Riad et al., 2021 [54]
60.	*Ericaria amentacea* (C. Agardh) Molinari & Guiry	SC-CO_2_ extraction; GC-MS	C_11_ unsaturated hydrocarbons were found in traces	Cikoš et al., 2022 [55]
61.	*Dictyopteris polypodioides* (A.P.De Candolle) J.V.Lamouroux		dictyopterene A (8.00%) dictyopterene C (0.87%) dictyopterene D (0.08%)	
62.	*Halopteris scoparia* (Linnaeus) Sauvageau	HS-SPME (divinylbenzene (DVB)/carboxene (CAR)/polydimethylsiloxane (PDMS) fibre); GC–MS	dictyopterene D (0.27–2.52%) dictyopterene C (0.92%)	Čagalj et al., 2022 [56]
		HS-SPME (PDMS/DVB fibre); GC–MS	dictyopterene D (0.35–1.23%) dictyopterene C (0.31%)	
		HD; GC–MS	dictyopterene D (0.06%) dictyopterene C (0.10–0.14%)	
63.	*Dasycladus vermicularis* (Scopoli) Krasser	HS-SPME; GC–MS	dictyopterene D (1.41–7.78%) dictyopterene C (8.65–9.34%)	Radman et al., 2022 [57]
		HD; GC–MS	dictyopterene D (0.17%) dictyopterene C (0.13%)	
64.	*Dictyota dichotoma* (Hudson) J.V. Lamouroux	HS-SPME (DVB/CAR)/PDMS) fibre); GC-MS	dictyopterene D (0.46–4.42%) dictyopterene C (0.46–1.03%)	Radman et al., 2022 [58]
		HS-SPME (PDMS/DVB fibre); GC-MS	dictyopterene D (0.07–8.22%) dictyopterene C (0.76–1.69%)	
		HD; GC-MS	dictyopterene C (0.04–0.13%)	
65.	*Cladostephus spongiosus* (Hudson) C. Agardh	HS-SPME with DVB/CAR/PDMS fibre; GC-MS	dictyopterene D (0.81–2.94%) dictyopterene C (0.49–1.3%)	Radman et al., 2023 [59]
		HS-SPME with PDMS/DVB fibre; GC-MS	dictyopterene D (0.44–1.34%) dictyopterene C (0.2%)	
		HD; GC-MS	dictyopterene A (0.07–0.08%) dictyopterene D (0.06–0.11%) dictyopterene (0.05%)	

## Data Availability

No new data were created or analyzed in this study. Data sharing is not applicable to this article.
